# 

**Published:** 1879-04

**Authors:** 


### CONTENTS.

PAGE

**CONTRIBUTIONS.**

Because Others Do, Ben. H. Pratt, ........ 115
Colds. P. H. Hayes, M. D ........ 118
Always Against the Woman. Part II. Ausburn Towner ........ 120
Child vs. Parent. Uncle Bob ........ 122

**MEDICAL ITEMS AND NEWS.**

Ether Jelley—Puritus—Facial Neuralgia—Epilepsy—Compound Bismuth Powder—Dysentery—Cancer—Gout—Expectorant—Phthisis—Iodoform—Croton Chloral Hydrate, Syrup—Febrifuge—Oxyures—Nitrate Silver Crayons—Anti-Emetic—Malerial Fever—Thymol—Antispasmodic—Villates Mixture in Sinuses—Diphtheria—Nitrate Amyl and Chloroform—Anginose Affection—Condurango—Gravel and Catarrh of Bladder—Tape-Worm—Antipyretic—Tape-Worm (2)—Salicylic Acid in Erysipelas—Chloral per Rectum—Hydrophobia—Chloral as Counter-Irritant—Phosphate of Zinc—Opium and Belladonna—Malarial Disorders—Diphtheria (2)—Opium Antidote—Morphia in Dysentery—Diarrhœa, Oxide of Zinc in—Skin Grafting—Urethral Fever—Apmorphia for Cleaning Œsophagus—Malarial Fevers—Yellow Fever—Urea in Blood—Counter-Irritation in Stings—Sugar Test—Acute Ophthalmia—Chronic Cough—Whooping-Cough—Nitrate Silver Ointment—Substitute for Ipecac ........ 124 to 134

PAGE

**OUR CORRESPONDENCE.**

Six Letter with Replies ........ 134 to 135

**HOUSEHOLD HINTS AND RECIPES.**

To Clean Carpets—Prepared Glue—Iridescent Glass—To Whiten Organ and Piano Keys—Smoky Ceilings, to Clean—To Preserve Fowers—White Wash—Steel Rust—Furniture Polish—Iron that will not Rust—Writing Pencils for Glass—Impressions from Prints ........ 136

**EDITORIAL.**

School Buildings ........ 137

Pedestrianism ........ 137

Disposal of the Dead ........ 138

**EDITORIAL CHIT-CHAT.**

The Cremation Society—Delinquents—Postage Stamps—Spring Flowers—John D. Williams—Dr. H. E. Reinhold—Dime Concerts—July Number—Chinese Bill—That Story of Fritz—Life-Saving Apparatus—City Architecture—Queer Dream—District Telegraph — Haights Hotel—Our Contributors—Eldridge Park—Col. Caldwell Cemeteries and Diphtheria—A Florist's Death—To the Profession—The Great English Physician Again—Fritz Visits the Conservatory ........ 139 to 142

